# The impact of comorbid depression in chronic rhinosinusitis on post-operative sino-nasal quality of life and pain following endoscopic sinus surgery

**DOI:** 10.1186/s40463-019-0340-0

**Published:** 2019-04-30

**Authors:** Javier Ospina, Guiping Liu, Trafford Crump, Jason M. Sutherland, Arif Janjua

**Affiliations:** 1 Division of Otolaryngology - Head and Neck Surgery, Sinus and Skull Base Surgery, Department of Surgery, University of British Columbia, Vancouver General Hospital, Vancouver, BC Canada; 2 Centre for Health Services and Policy Research, School of Population and Public Health, University of British Columbia, Vancouver, BC Canada; 3 Department of Surgery, University of Calgary, Calgary, Alberta Canada; 4 Rhinology, Endoscopic Sinus and Skull Base Surgery, Division of Otolaryngology - Head and Neck Surgery, Department of Surgery, University of British Columbia, Vancouver, BC Canada

**Keywords:** Chronic rhinosinusitis, Endoscopic sinus surgery, Quality of life, Symptom severity, Patient-reported outcomes

## Abstract

**Background:**

Depression and chronic pain are debilitating disorders that co-exist with many chronic diseases. Chronic rhinosinusitis (CRS) is no exception. Nonetheless, little is known about the association between these co-related conditions and the treatment of CRS. The objective of this study is to measure outcomes following endoscopic sinus surgery (ESS) in CRS patients reporting significant pre-operative depression and pain.

**Methods:**

This is a prospective longitudinal cohort study examining patients with CRS who had failed maximal medical therapy and subsequently underwent ESS. Participants completed a several patient-reported outcome (PRO) instruments pre-operatively and 6 months post-operatively. The PROs included the Sinonasal Outcome Test-22 (SNOT-22), the Patient Health Questionnaire (PHQ-9) measuring symptoms of depression and an assessment of chronic pain using the pain intensity (P), interference with enjoyment of life (E) and general (G) activity instrument, the PEG instrument.

**Results:**

The study had 142 participants complete their pre-operative and post-operative surveys. The participation rate was 40.1% among eligible patients. The prevalence of at least moderate depression was 22 patients (15.5%) among participants. Compared with non-depressed participants, the pre-operative sino-nasal disease burden and pain scores were higher among depressed participants (*p* <  0.001) and the gain in health following surgery was smaller (*p* <  0.001).

**Conclusions:**

Pre-operative disease burden is higher among depressed patients. Post-operative gains in sino-nasal quality of life attributable to endoscopic sinus surgery were significantly smaller among depressed participants. Pre-operative screening for depression could identify opportunities for medical intervention and improve outcomes among CRS patients.

## Background

Chronic rhinosinusitis (CRS) is a frequently occurring disease, with significant impact on quality of life and health care spending. It is believed that CRS affects at minimum 5 % of Canadians [1] and 14 to 16% of the U.S. population [2]. Patients with CRS report as much or more bodily pain and worse social functioning than patients with other chronic conditions such as chronic obstructive pulmonary disease, congestive heart failure, and back pain [3].

CRS patients commonly present with symptoms of fatigue, frustration, and physical pain that may be confounded with, or compounded by, the effects of an underlying depressive disorder [4]. Recent publications have shown that the prevalence of depression in patients with CRS is estimated to be in the range of 20–25% [4–6].

The role of the dyad of pain and depression in CRS has recently gained major interest in understanding this complex disease. This interplay, and it’s influence on treatment outcomes, has yielded conflicting results; some authors have shown no difference in post-operative outcomes among depressed and non-depressed patients, while others have demonstrated that depressed patients don’t experience the same gains in health [4, 5, 7–9].

Since CRS is a condition whose constellation of symptoms has not correlated well with objective outcome measures, such as nasal endoscopy or imaging [10–12], this study focuses on post-operative outcomes measured via validated patient-reported outcome (PRO) instruments. There is evidence that PROs have been effective at accurately measuring ESS outcomes among patients with CRS [13].

This research provides significant clinical relevance in surgical decision making for CRS patients who may be psychologically affected. This subgroup of the CRS patient population warrants special attention considering that surgery alone, as treatment, might not offer the expected degree of improvement for these patients.

The objective of this study is to contribute to the understanding of the role of depression among CRS patients treated with ESS. Clarifying the interplay between depression and ESS outcomes is clinically relevant since surgeons need to decide whether to operate on patients who demonstrate significant symptoms of depression. This sub-group of CRS patients warrants attention since surgery alone might not offer sought-after outcomes.

## Methods

Persistently symptomatic CRS patients who fail appropriate medical therapy under the care of a rhinologist, and subsequently booked for ESS, are prospectively identified from two dedicated rhinology subspecialty practices in Vancouver, Canada. The 2015 Canadian Sinusitis Guidelines were used to establish the diagnosis of CRS [14]. Inclusion criteria were patients 19 years or older, who were are able to respond to survey questions in English with or without the assistance of a translator. Eligible patients are contacted up to twice by phone to participate within 2 weeks of being scheduled for ESS. Patients who agree to participate receive a survey package either through the mail or by email to complete a web-based version. The survey packages includes study description, study personnel contact information, a number of PRO instruments and a stamped return envelope.

This study includes pre-operative and post-operative data collection initiated in September 2012 concluding in July 2017. The University of British Columbia’s Behavioural Research Ethics Board (BREB) approved the study.

### Survey instruments

The Sino-nasal Outcome Test-22 (SNOT-22) is a rhinology-specific quality of life scale commonly used to measure outcomes among CRS participants [15]. The SNOT-22 includes 22 items, including symptoms of nasal obstruction, smell disturbances and sleep quality. Each item is scored from one to five, and a lower score is associated with better sino-nasal quality of life. Evaluations of the SNOT-22 have concluded that the instrument’s properties include reliability, validity, and responsiveness [13], though recent research has found that the items measuring mental health do not perform well [16]. The Minimal Important Difference (MID) for the SNOT-22 test is 8.9 points - thus a change of less than 9 points cannot be perceived as a significant clinical improvement by the patient [15].

Participants’ symptoms of depression were measured with the Patient Health Questionnaire (PHQ-9) [17]. A number of studies have demonstrated that the PHQ-9 has good internal consistency and test-retest reliability, strong criterion and construct validity in a number of clinical settings [18, 19]. This nine item instrument measures participants’ level of depression in two domains: symptoms and functional impairment [17] and whose recall period is the past 2 weeks. All items’ scores are summed to generate the overall PHQ-9 score. Scores greater than, or equal to, 10 have been considered clinically significant depression, while scores greater than 20 represent severe depression [17].

Participants’ pain was measured using the pain intensity (P), interference with enjoyment of life (E) and general (G) activity instrument – PEG [20]. This brief instrument includes one pain intensity item and two pain interference items and whose recall period is the past week. The overall score is the average of the three items. Scores greater than three are indicative of high pain [21]. The PEG has demonstrated responsiveness to change in pain [22], strong intra-rater reliability, and good validity with the Brief Pain Inventory (BPI) [20].

### Analysis

Participants are stratified into two groups according to pre-operative depression. Stratification into sub-groups representing non-depressed and depressed is defined by pre-operative PHQ-9 scores of less than 10 [17]. For both groups, participants’ SNOT-22 scores are summarized by demographic subgroups.

Participant’s sino-nasal symptom severity are stratified into quartiles based on pre-operative SNOT-22 scores. Improvements in sino-nasal symptoms (i.e., change in SNOT-22 score) is analyzed for the non-depressed and depressed sub-groups using analysis of variance methods. Participant’s PEG scores are stratified into quartiles based on pre-operative PEG scores. The change in PEG scores following ESS are shown for the non-depressed and depressed sub-groups. The analyses does not correct for multiple comparisons and *p*-values are unadjusted. There were no missing values in the data. Anonymized data is analyzed using SAS 9.4 (SAS, Cary, NC).

A linear regression model is used to measure the patients’ change in SNOT-22 scores after surgery adjusting for demographic characteristics of sex, age and presence of depression. The primary hypothesis is whether the gain in SNOT-22 scores will be different between the non-depressed and depressed sub-groups (two-sided test). The least square means of the sub-group differences are calculated. The models fit is evaluated by examination of residuals. Regression coefficients, standard errors and *p*-values are calculated.

## Results

There were 354 eligible patients, among whom 57.2% agreed to participate and completed the preoperative survey. Non-participants were, on average, 3 years younger than participants, though no other demographic differences were found between participants and non-participants. Among participants that completed the preoperative survey, 70.1% completed the postoperative survey, providing the study with an overall participation rate among eligible patients of 40.1% and 142 participants. There were no differences in participants’ preoperative PHQ-9 scores between participants who completed the postoperative survey with those participants that did not complete the postoperative survey.

Among participants, there were 22 participants (15.5%) with CRS whose PHQ-9 scores was 10 or greater, indicating clinically significant depression. As shown in Table 1, the mean pre-operative SNOT-22 scores was significantly higher among depressed participants (59.8) than non-depressed (36.4) participants (*p* <  0.001).

**Table 1:** Summary statistics of patients’ pre-operative SNOT-22 score, age group and sex categories; stratified by depressed and non-depressed patients

	All Participants			Non-depressed Participants PHQ-9 < 10			Depressed Participants PHQ-9 ≥ 10		
	N	Mean	SD	N	Mean	SD	N	Mean	SD
*Overall*	*142*	*40.0*	*20.8*	*120*	*36.4*	*19.3*	*22*	*59.7*	*15.2*
Age Group									
≤ 40	20	36.7	17.5	17	34.7	18.1	3	48.0	9.0
41–55	43	47.6	23.1	34	42.6	21.5	9	66.4	19.9
56–70	59	39.3	19.5	51	36.5	18.8	8	57.3	14.3
70 +	20	28.9	16.5	18	25.7	12.5	2	57.5	26.2
Sex									
Female	62	43.4	21.5	38.1	18.3	38.1	11	68.3	17.6
Male	80	37.3	19.9	35.1	20.0	35.1	11	51.3	12.8

**Fig. 1:**
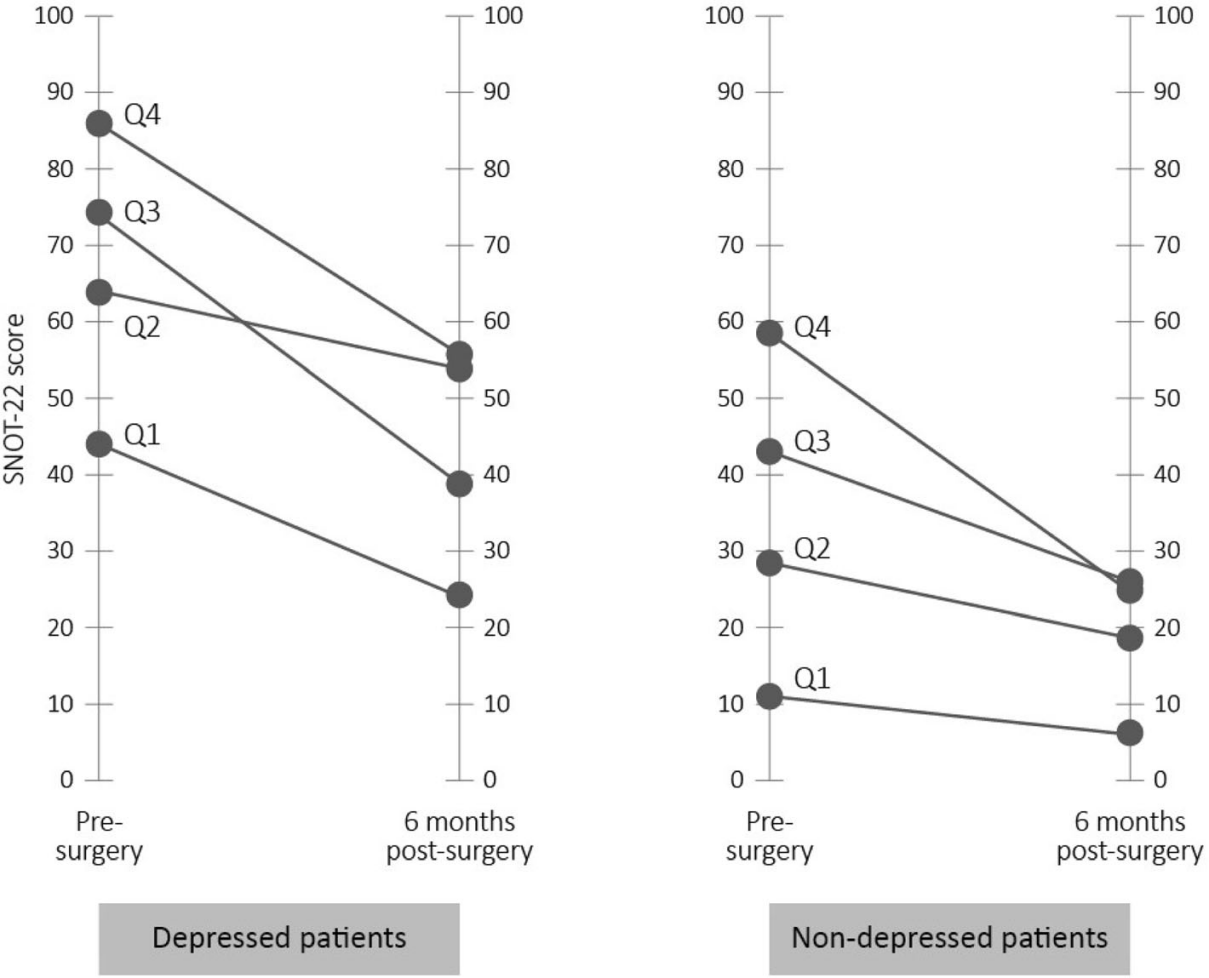
Comparison of pre- and post-operative SNOT-22 scores among non-depressed and depressed sub-groups of participants

As shown in Fig. 1, regardless of the presence of depression, participants with SNOT-22 scores in all quartiles tended to report improvements in SNOT-22 scores. Participants reporting the highest pre-surgical SNOT-22 scores experienced the largest gains in sino-nasal quality of life. For additional detailed data, see Appendix 1.

As shown in Table 2, the regression analysis results show that, adjusting for demographic characteristics and pre-operative SNOT-22 score, participants with pre-operative depression gain, on average, 14 points less than non-depressed participants (*p* <  0.001). The least squares mean gain in SNOT-22 score for non-depressed participants was 19.5 and 5.2 for depressed participants (*p* < 0.001).

**Table 2:** Factors associated with change in participants’ SNOT-22 score, adjusting for demographic characteristics and pre-operative SNOT-22 score

Regression Effect	Coefficient Estimate	Standard Error	*P*-Value
Intercept	4.45	4.23	0.29
Age Group			
≤ 40	Reference		
41–55	−4.50	3.91	0.25
56–70	− 0.91	3.69	0.81
70 +	6.93	4.53	0.18
Sex			
Female	Reference		
Male	−0.48	2.44	0.84
Pre-operative SNOT-22 score	−0.60	0.07	< 0.001
Depression Sub-group (Pre-operative)			
Not depressed	Reference		
Depressed	14.28	3.63	< 0.001

**Fig. 2:**
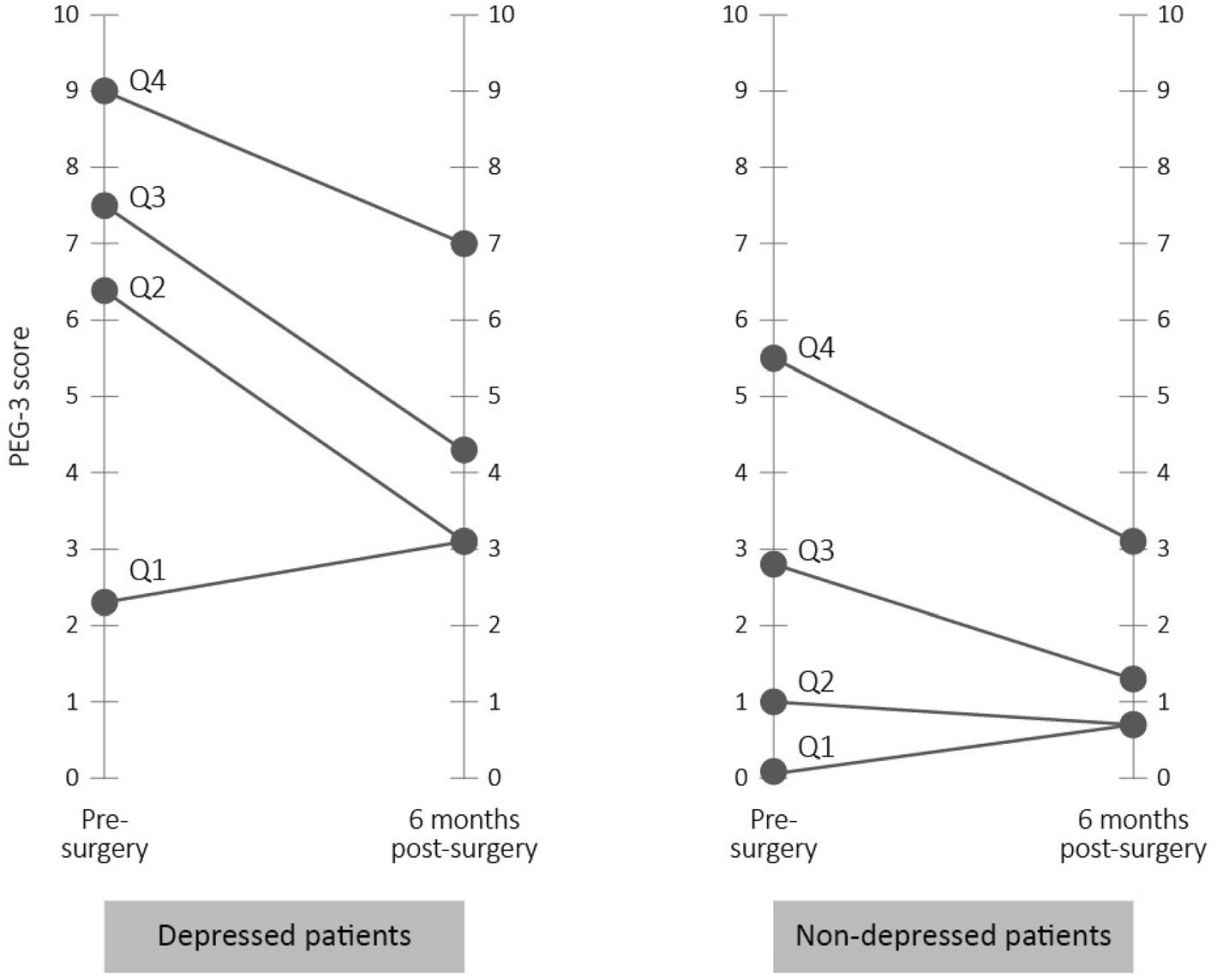
Sub-group comparison of PEG pain scores among non-depressed and depressed participants

As shown in Fig. 2, pains scores improved among the depressed and non-depressed sub-groups. Among depressed participants, those within the highest quartile of pre-operative pain, PEG scores improved from 8.7 to 7.1. Among non-depressed participants with the highest pre-operative pain, PEG scores improved from 5.9 to 2.2. Of note, depressed participants reporting severe pre-operative pain did not improve scores to the level of non-depressed participants. For additional detailed data about PEG scores, see Appendix 2.

## Conclusions

Depression and chronic pain are two of the most debilitating disorders in our population, limiting quality of life and employment opportunities. These comorbid conditions appear to occur in clinical settings with extremely high prevalence, especially in patients with chronic levels of inflammation.

Prevalence of depression (measured with PHQ-9) among our participants with subsequent surgically-treated CRS (15.5%) was consistent with previous reports, estimated to range between 20 and 25% [4–6]. Other authors have used different screening tools, such as the Beck Depression Inventory (BDI) to identify comorbid depression in CRS patients, showing an overall prevalence as high as 31% [23]. A recent publication has shown that depression-associated symptoms are the driving factor for missed days of work or school in individuals with CRS [24]. In our study, pre-operative SNOT-22 scores were significantly higher among depressed participants, illustrating worse disease-specific quality of life in this subgroup.

Chronic inflammation can cause a cascade of inflammatory mediators. This effect can lead to adaptive normal responses (fatigue, enhanced pain sensitivity, neurovegetative symptoms, sleep disorders) that is seen in acute inflammation, as well as chronic global health conditions, such as depression and ongoing pain [25]. Since these symptoms can persist after the inflammation has subsided, treatment for chronic depression and pain may be instrumental in improving health-related quality of life.

Although the pathophysiologic mechanisms through which depression and pain affect the quality of life in CRS patients is not completely understood; the literature supports our presumption that inflammation contributes, via some established mechanistic pathways [26]. Meta-analytic results have shown significantly higher concentrations of the pro-inflammatory cytokines TNF-α and IL-6 in depressed subjects compared with control subjects [27], strengthening the evidence that depression is accompanied by activation of the inflammatory response system.

These inflammation pathways may play a key role in the pathophysiology of depression especially among women, given that women show higher levels of inflammation and higher rates of autoimmune diseases compared to men [28]. These links between sex, inflammation and depression might explain why our study found higher preoperative SNOT-22 scores in women compared with males within the depressed patient cohort. These findings suggest that clinicians should pay special attention to women with CRS, since they experience worse disease-specific health-related quality of life and may have more susceptibility to inflammation and depression.

This study showed high levels of pain in preoperatively depressed participants compared to non-depressed participants. Similarly to other studies that have shown the dyad of pain and depression are found to coexist in CRS patients [29], our data further supports the complex interrelation between the two entities and CRS. While studies have shown the importance of understanding this interplay, there is a dearth of available literature regarding optimal pathways of treatment for patients with these comorbidities.

Endoscopic sinus surgery is the preferred surgical strategy for CRS among patients with medically refractory CRS [13, 30, 31]. However, it is well known that some patients do not respond equally well to this treatment strategy and warrant further research. The role of depression is yet ill-understood; however, our data supports that depressed patients are likely to experience much smaller gains in health, an explanation further grounded in other research showing patients found suffering from depression have poorer disease-specific and general quality of life outcomes after surgery [5, 7, 32]. However, it is unclear whether treating depression and pain with other treatment modalities would improve post-operative CRS outcomes.

There are clinical application of our findings. First, clinicians should identify depression as a comorbid condition, as this may significantly influence postoperative CRS outcomes. We propose, and have begun to implement, utilizing a validated depression tool in pre-operative surgical patients. Secondly, while several studies have shown significant symptom improvement among patients with the most severe sino-nasal symptoms, our findings suggest that the magnitude of this improvement may not be as significant among depressed patients. This study also clearly raises the question about the role of pre-operative treatment of depression, as an adjunct to the medical and surgical of CRS.

There are limitations to be considered with this study. First, we failed to differentiate between CRS with nasal polyposis (CRSwNP) and CRS without nasal polyposis (CRSsNP) in our population. Other investigators have recently found that CRSsNP patients may have an increased prevalence of depression, compared to healthy controls. Depressed patients can also have more severe pain, whereas CRSwNP present with more severe symptoms of nasal congestion and olfactory loss [23]. This phenotypic differentiation could be useful for further sub-group analysis. Second, we did not measured the objective burden of the disease with other objective tools such as Lund-Mackay scoring system and Lund-Kennedy endoscopic score. This would allow us to measure potential correlation between the severity of clinical and radiologic manifestations of CRS, and the severity of pain and depression in this patient population. Finally, our follow-up after surgery was only 6 months. Studies with longer follow-up analysis may help determine whether the magnitude of the change achieved in QoL, depression and pain after surgical intervention in CRS patients persist in the long-term after surgery.

Depression, pain and chronic rhinosinusitis are common coexisting conditions associated with poorer post-operative ESS outcomes among CRS patients. Clinicians should be aware that ventilation and local control of inflammation via standard medical and surgical treatments for CRS may be not sufficient for depressed patients. A more comprehensive understanding of this complex interplay and the treatment strategies that could be used to ameliorate the effect of depression on postoperative health outcomes warrant further investigation.

## Appendix 1

Comparison of pre-operative and post-operative sino-nasal symptoms (SNOT-22) scores among depressed and non-depressed participants.

**Table 3:** SNOT 22 values among depressed patients (PHQ-9 ≥ 10)

SNOT-22 Score at baseline - Quartiles	Pre-Op SNOT-22 Score			Post-Op SNOT-22 Score		
	N	Mean	Median	N	Mean	Median
0-25th Q	6	38.2	39	6	13.8	13.5
26-50th Q	5	53.6	54	5	30.6	30.0
51-75th Q	6	68.2	68	6	61.8	63.0
76-100th Q	5	81.8	83	5	57.6	61.0

**Table 4:** SNOT 22 values among non-depressed patients (PHQ-9 < 10)

SNOT-22 Score at baseline - Quartiles	Pre-Op SNOT-22 Score			Post-Op SNOT-22 Score		
	N	Mean	Median	N	Mean	Median
0-25th Q	30	11.8	12	30	9.2	10
26-50th Q	31	28.9	29	31	19.0	15
51-75th Q	31	44.2	43	31	22.1	18
76-100th Q	28	62.1	61	28	21.7	19

## Appendix 2

Comparison of pre- and post-operative PEG pain scores among depressed and non-depressed participants.

**Table 5:** PEG values among depressed patients (PHQ-9 ≥ 10)

PEG-3 Score at baseline - Quartiles	Pre-Op PEG-3 Score			Post-Op PEG-3 Score		
	N	Mean	Median	N	Mean	Median
0-25th Q	6	1.1	1.3	6	1.7	0.5
26-50th Q	5	4.8	5.0	5	2.5	2.0
51-75th Q	6	6.8	6.8	6	5.6	6.2
76-100th Q	5	8.7	9.0	5	7.1	8.3

**Table 6:** PEG values among non-depressed patients (PHQ-9 < 10)

PEG-3 Score at baseline - Quartiles	Pre-Op PEG-3 Score			Post-Op PEG-3 Score		
	N	Mean	Median	N	Mean	Median
0-25th Q	35	0.1	0.0	35	0.6	0.0
26-50th Q	34	1.3	1.0	34	1.1	0.7
51-75th Q	23	3.1	3.0	23	1.4	0.7
76-100th Q	28	5.9	6.0	28	2.2	1.0

## Abbreviations

BREB: Behavioural Research Ethics Board
CRS: Chronic rhinosinusitis
CRSwNP: CRS with nasal polyposis
ESS: Endoscopic sinus surgery
PRO: Patient-reported outcome
SNOT-22: Sinonasal Outcome Test-22
